# Determination and Analysis of the Putative AcaCD-Responsive Promoters of Salmonella Genomic Island 1

**DOI:** 10.1371/journal.pone.0164561

**Published:** 2016-10-11

**Authors:** Gábor Murányi, Mónika Szabó, Ferenc Olasz, János Kiss

**Affiliations:** Agricultural Biotechnology Institute, National Agricultural Research and Innovation Centre, Gödöllő, Hungary; University of Manchester, UNITED KINGDOM

## Abstract

The integrative genomic island SGI1 and its variants confer multidrug resistance in numerous S*almonella enterica* serovariants and several *Proteus mirabilis* and *Acinetobacter* strains. SGI1 is mobilized by the IncA/C family plasmids. The island exploits not only the conjugation apparatus of the plasmid, but also utilizes the plasmid-encoded master regulator AcaCD to induce the excision and formation of its transfer-competent form, which is a key step in the horizontal transfer of SGI1. Triggering of SGI1 excision occurs via the AcaCD-dependent activation of *xis* gene expression. AcaCD binds in P_*xis*_ to an unusually long recognition sequence. Beside the P_*xis*_ promoter, upstream regions of four additional SGI1 genes, *S004*, *S005*, *S012* and *S018*, also contain putative AcaCD-binding sites. Furthermore, SGI1 also encodes an AcaCD-related activator, FlhDC_SGI1_, which has no known function. Here, we have analysed the functionality of the putative AcaCD-dependent promoter regions and proved their activation by either AcaCD or FlhDC_SGI1_. Moreover, we provide evidence that both activators act on the same binding site in P_*xis*_ and that FlhDC_SGI1_ is able to complement the *acaCD* deletion of the IncA/C family plasmid R16a. We determined the transcription start sites for the AcaCD-responsive promoters and showed that orf *S004* is expressed probably from a different start codon than predicted earlier. Additionally, expression of *S003* from promoter P_*S004*_ was ruled out. P_*xis*_ and the four SGI1 promoters examined here also lack obvious -35 promoter box and their promoter profile is consistent with the class II-type activation pathway. Although the role of the four additionally analysed AcaCD/FlhDC_SGI1_-controlled genes in transfer and/or maintenance of SGI1 is not yet clear, the conservation of the whole region suggests the existence of some selection for their functionality.

## Introduction

Conjugative genomic islands (GIs) have a key role in rapid dissemination of multidrug resistance (MDR) in *Salmonella* and other Gram-negative pathogenic bacteria. *Salmonellae*, particularly several serovars such as Typhimurium and Enteritidis, are among the most prevalent zoonotic pathogens worldwide. The MDR clone of *S*. Typhimurium DT104 (resistant for ampicillin, chloramphenicol/florfenicol, streptomycin/spectinomycin, sulphonamides and tetracycline—ACSSuT phenotype) emerged in the early 1980s in the UK and became globally distributed in the 1990s among humans and livestock [1]. The Salmonella genomic island 1 (SGI1) [2], a 42.4-kb chromosomal gene cluster, turned out to be responsible for the MDR phenotype of DT104 clones [3]. So far, numerous SGI1 variants showing different resistance patterns have been described mostly from *S*. *enterica* serotypes [4–10], from several *Proteus mirabilis* strains [11, 12] and recently from *Acinetobacter baumannii* strains [13], posing significant challenges for clinicians in human and animal healthcare.

**Fig 1:**
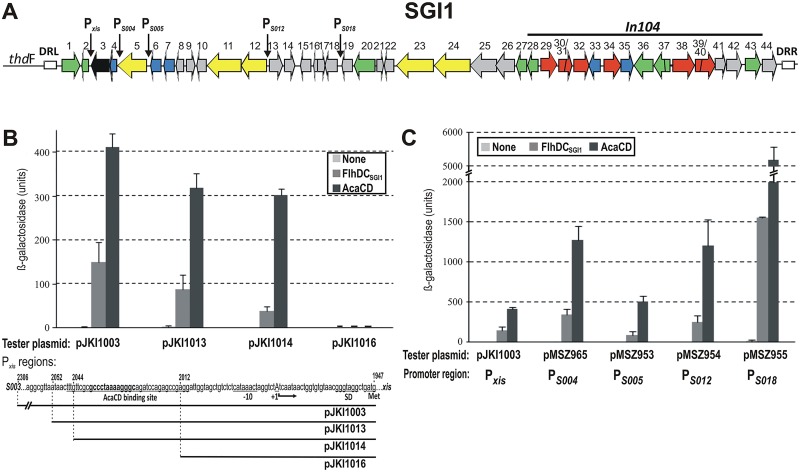
Induction of the predicted AcaCD-dependent promoters of SGI1. (A) The schematic map of SGI1. The predicted AcaCD-dependent promoters are indicated by vertical arrows. Orfs *S001-S044* represented by horizontal arrows are numbered, the complex integron region In104 is indicated above the graph. Left and right direct repeats are shown as white boxes. Color code of gene functions: green—recombination, black—replication, blue—regulator, yellow—conjugation, red—antibiotic resistance, grey—unknown. (B) Determination of the target site of FlhDC_SGI1_ activator in P_*xis*_ promoter region. The graph shows the promoter activities of the truncated P_*xis*_ regions measured by the β-galactosidase assay in the presence of FlhDC_SGI1_ or AcaCD. Coordinates and sequence of the promoter region in the tester plasmids are shown below the graph. The functional domains of P_*xis*_ determined previously are underlined and indicated below the sequence, the conserved core of AcaCD-binding site is shown as bold, TSS is marked with uppercase. (C) Activation of five SGI1 promoters containing predicted AcaCD-binding site by FlhDC_SGI1_ and AcaCD. The activators were expressed from p15A-based producer plasmids pGMY6 and pJKI888, while the empty vector pJKI88 was applied as negative control.

SGI1 was described as an integrative mobilizable element (IME) [14], residing in the 3’ end of the *thdF* gene (Fig 1A) that is efficiently and exclusively mobilized by the IncA/C family conjugative plasmids [10, 15, 16]. The SGI1 prototype carries 44 predicted open reading frames (orfs) and is delimited by imperfect 18-bp direct repeats DRL and DRR. The well conserved backbone consists of the first 27 and the last orfs, interrupted by a complex integron region In104 that includes some mobile elements and the resistance genes associated with the ACSSuT phenotype [17]. Unlike the integrative conjugative elements, such as SXT-family members, SGI1 does not encode a complete gene set of the conjugation apparatus. Although the backbone carries several conjugation-related genes, these are dispensable for conjugation [10] and the horizontal transfer of SGI1 depends on the IncA/C helper plasmids.

The only self-encoded proteins proved to be required for SGI1 transfer are the lambda integrase family member Int and the recombination directionality factor Xis, which are encoded near the 5’ end of the island [14, 18]. Expression of *int* is constitutive, while *xis* is activated by the FlhDC-family regulator AcaCD encoded by the IncA/C plasmids [16, 18]. Excision catalysed by Int and Xis produces the free, mobilizable form of SGI1. On the other hand, the site-specific chromosomal integration required for vertical transmission is carried out solely by Int. As Int can excise SGI1 inefficiently without Xis [14], its activity is pushed towards integration and contributes to the high stability of SGI1 in the absence of helper plasmids [10]. Due to the AcaCD-dependent control of *xis* expression, the helper entry triggers SGI1 excision, which is a key aspect in hijacking the conjugation system of the IncA/C helper plasmids [18].

AcaCD is an FlhDC-family master activator of all transfer-related genes in IncA/C plasmids [16]. The activator has been shown to act on unusually long binding sites that share a well conserved core motif (GCCCAAAATGGGC). Using the consensus sequence of plasmid-borne AcaCD binding sites, five putative target sites upstream of the genes *xis*, *S004*, *traN (S005)*, *traH (S012)* and *S018* have been predicted on SGI1 [16]. Although the regulatory effect and binding of AcaCD to the predicted site in the *xis* promoter region have been reported [18], the other four putative AcaCD-driven promoters have not yet been examined in detail. Furthermore, an *flhDC* homologue gene closely related to *acaCD*, named as *flhDC*_*SGI1*_, has been identified on SGI1. Excess FlhDC_SGI1_ also promotes SGI1 excision, but has a weaker destabilization effect on the island compared to AcaCD [18]. Protein sequence similarities and the activity on SGI1 suggest that AcaCD and FlhDC_SGI1_ can act on the same target sites.

In this study, we analyse the SGI1 promoters that have previously been predicted to be controlled by AcaCD. Here we report that all the five promoters are activated by either AcaCD or FlhDC_SGI1_, however, the latter has weaker effect. It is also shown that both regulators act on the same binding site in P_*xis*_ and that FlhDC_SGI1_ is able to complement the *acaCD* deletion of the IncA/C family plasmid R16a. We determined the transcription start site (TSS) for the AcaCD-responsive promoters and showed that orf *S004* is probably expressed from a different start codon than predicted in the annotation of SGI1 orfs and this shorter S004 peptide has inhibitory effect on bacterial growth. Contrary to previous assumptions [16], we provide evidence that the expression of *repA* (*S003*) is regulated independently of AcaCD.

## Materials and Methods

### DNA and microbial techniques

Standard molecular biology techniques were carried out according to [19]. Oligonucleotide primers used are listed in S1 Table. Primers annealing to SGI1 were designed according to the sequence GenBank: AF261825.

Bacterial strains (listed in S2 Table) were routinely grown at 37°C in LB broth supplemented with the appropriate antibiotics in the final concentration of: ampicillin (Ap) 150 μg/ml, chloramphenicol (Cm) 20 μg/ml, kanamycin (Km) 30 μg/ml, spectinomycin (Sp) 50 μg/ml, streptomycin (Sm) 50 μg/ml, nalidixic acid (Nal) 20 μg/ml, gentamicin (Gm) 25 μg/ml, tetracycline (Tc) 10 μg/ml.

For the complementation tests, plasmids pJKI1038 (AcaCD-producer), pJKI1040 (FlhDC_SGI1_-producer) and pJKI1036 (negative control) were transformed into TG1Nal::SGI1-C/R16aΔ*acaCD* strain [18] and five transformant colonies were used as donors in conjugation with TG90 recipient. Conjugation assays and calculation of transconjugant frequencies were performed as described previously [10] except that crosses were incubated for 6 hours. In this experiment, SGI1-C variant [20], a spontaneous deletion derivative of SGI1 retaining only the Sm^R^/Sp^R^ and Sul^R^ markers, was applied due to its simple resistance phenotype. SGI1-C has an intact backbone and its mobilization does not differ from that of SGI1 prototype [10].

To detect proteins encoded by orfs *S003*, *S004* (designated as *S004*_L_) and the shorter version of *S004* (called *S004*_S_), total protein samples were purified from non-induced and induced (0 or 0.5 mM IPTG, respectively) *E*. *coli* strain Tuner(DE3) (Novagen) harbouring the expression plasmids pJKI1048, pJKI1049 or pJKI1050, respectively. After 3 hours of incubation under vigorous shaking at 37°C, total protein samples were purified according to [19] and run on 12.5% (S003) or 15% (S004_L_ and S004_S_) SDS PAGE gels.

The growth curves for plasmid bearing strains were determined as follows: six transformant TG1Nal colonies harbouring pJKI1048, pJKI1049, pJKI1050 or pGMY8 (negative control) were grown overnight in 2 ml LB+Nal+Sm+Sp broth. Cultures were diluted 1:200 in 12 ml LB+Nal+Sm+Sp supplemented with 0.05 mM IPTG, grown at 37°C under vigorous shaking and OD_600_ was measured every 30 min for five hours and after 24 hours.

### Plasmid constructions

Relevant features of plasmids are listed in S3 Table. For step-by-step plasmid constructions see S1 Text.

### β-galactosidase assay

β-galactosidase assays were performed as described [18]. Briefly, the tester constructs pJKI1003 (P_*xis*_), pJKI1013, pJKI1014, pJKI106 (truncated P_*xis*_ regions), pMSZ965 (P_*S004*_), pMSZ953 (P_*S005*_), pMSZ954 (P_*S012*_) and pMSZ954 (P_*S018*_) and one of the producer plasmids expressing AcaCD (pJKI888) or FlhDC_SGI1_ (pGMY6) or pJKI88 as negative control were transformed into TG1 cells. Transformant colonies were grown overnight under selection for both plasmids in 2 ml LB+Km+Ap, the overnight cultures were diluted 40×, induced with 0.05 mM IPTG and grown ca. OD_600_ = 0.3. β-galactosidase assay were then carried out according to [21].

### Primer extension analysis

Total RNA was isolated from TG1 strain containing the AcaCD-producer plasmid pJKI888 (AcaCD+) or pJKI88 as negative control (AcaCD-) with one of the tester plasmids pMSZ965 (P_*S004*_), pMSZ953 (P_*S005*_), pMSZ954 (P_*S012*_) and pMSZ954 (P_*S018*_), respectively. Overnight cultures were diluted 1000×, supplemented with 0.5 mM IPTG to induce transcription of the activator and grown to an OD_600_ of 0.6 in 10 ml LB without antibiotics at 37°C. Cells were harvested from 2.5 ml cultures and frozen in liquid nitrogen. 750 μl of lysis buffer (0.2 M Na-acetate pH 5.2, 1% SDS 10 mM EDTA) was added to the frozen pellet. After vortexing the mix was boiled for 2–3 min and then vortexed again for 2 min. Lysates were centrifuged (20 min, 16000 rcf, 4°C) and the supernatants were extracted with 750 μl phenol, and centrifuged again (10 min, 16000 rcf, 4°C). Extraction was repeated with 720 μl phenol:chloroform (1:1), then with 360 μl chloroform. RNA was precipitated by adding 200 μl 10 M LiCl to 600 μl supernatant followed by incubation on ice (1 hour) and centrifugation (10 min, 16000 rcf, 4°C). Pellet was first washed with 2.5 M LiCl, then with 70% ethanol, centrifuged, air dried and dissolved in 50 μl RNase-free water at 50°C. 20 μl (~10 μg) of total RNA was digested with 50 units of RNase-free DNaseI (Qiagen) in a final volume of 50 μl (10 min, 37°C), then the enzyme was inactivated (10 min, 65°C). The reaction mix was phenol-chloroform extracted, washed twice with 70% ethanol and dissolved in 20 μl RNase-free water after drying. RNA concentration was set to 0.5 μg/μl using NanoDrop1000 Spectrophotometer (Thermo Scientific). To control the quality 1 μg of each RNA sample was run on a 1% TAE agarose gel.

Primer extension assay was performed using RevertAid H Minus first strand cDNA synthesis kit (Fermentas). One μl of pUCfor21 primer (10 μM) was labelled in 10 μl volume with 10 units of polynucleotide kinase (Fermentas) using 50 μCi [γ-^32^P] dATP (45 min, 37°C) followed by inactivation of the enzyme (10 min, 68°C). Approximately 5 μg of RNA and 2 pmol of ^32^P-labeled primer were mixed, heated to 70°C for 5 min, and then allowed to anneal at 37°C for 5 min. Extension reactions were carried out in RT buffer (50 mM Tris-HCl [pH 8.3], 50mM KCl, 4 mM MgCl_2_ 10 mM DTT) with 1 mM dNTP and 20 u RiboLock ribonuclease inhibitor in a total volume of 20 μl (42°C, 60 min) using 200 units of reverse transcriptase. Extension products were precipitated by ethanol, resuspended in 3 μl DEPC-treated water, and combined with 2 μl sequencing loading solution. The sequence ladder for the tester plasmids was generated with primer pUCfor21 using a Sequenase version 2.0 DNA sequencing kit (USB) according to the manufacturer’s protocol. The products of each reaction were electrophoresed on a 6% denaturing polyacrylamide gel at 1800V. The gel was exposed to storage phosphor screen and scanned on Storm 840 PhosphorImager (Amersham Biosciences).

## Results and Discussion

### FlhDC_SGI1_ and AcaCD act on the same target sites

Excess FlhDC_SGI1_, the SGI1-encoded FlhDC-family master activator, has been previously shown to promote the excision and frequent loss of SGI1 [18], however, its target site was not determined. The closely related AcaCD regulator, which is encoded by IncA/C plasmids, has similar but somewhat stronger effect on SGI1. As AcaCD has been shown to bind to the P_*xis*_ promoter region 61–98 bp upstream of the start codon of *xis* gene [18] we supposed that FlhDC_SGI1_ could also act on the same target site. To test this assumption, β-galactosidase assay was carried out to compare the activation effect of AcaCD and FlhDC_SGI1_ on different parts of P_*xis*_ that were inserted into tester plasmids containing the *lacZ* gene without its own promoter [18]. The tester plasmid pJKI1003 carried the whole non-coding region between *xis* and *S003* (P_*xis*_ region). pJKI1013 contained the proximal part of P_*xis*_ with the intact AcaCD binding site along with several flanking bases, while pJKI1014 carried the shortest part of P_*xis*_ that previously proved to be fully active with AcaCD. On the other hand, pJKI1016 containing the proximal part of P_*xis*_ along with the last three bp of the AcaCD-protected region but without the core AcaCD binding site, cannot be activated by AcaCD (Fig 1B). Thus, pJKI1014 and pJKI1016 differs only in the presence or absence of AcaCD binding site [18].

AcaCD and FlhDC_SGI1_ were expressed from analogous producer plasmids pJKI888 and pGMY6, respectively, and their activating effects on the above promoter fragments were measured. Both activators induced *lacZ* expression only in case of the three constructs carrying AcaCD binding site, while no *lacZ* expression was detected with the tester plasmid pJKI1016 lacking the binding site. FlhDC_SGI1_ appeared to be less efficient activator than AcaCD, showing only 13–36% of activity on compared to its plasmid-borne counterpart (Fig 1B). The results, nevertheless, proved that FlhDC_SGI1_ and AcaCD act on the same target site in P_*xis*_ region.

The relatively high similarity between the two activators (89% identity for C and 67% for D subunits) [18] and the fact that they have a common target site in P_*xis*_ suggested that FlhDC_SGI1_ can act on other AcaCD binding sites, as well. It was previously demonstrated that deletion of *acaCD* abolished the conjugation of IncA/C plasmids and could be complemented by AcaCD expression in *trans* [16][18]. Accordingly, the derivative of the IncA/C plasmid R16a that bears an *acaCD* knock-out (R16aΔ*acaCD*) was used to test whether FlhDC_SGI1_ can substitute for the missing AcaCD. The complementation test using plasmids that express AcaCD or FlhDC_SGI1_ (pJKI1038 and pJKI1040, respectively) showed that FlhDC_SGI1_ could activate the transfer system of R16a in the absence of AcaCD and promoted the conjugation of both the mutant R16aΔ*acaCD* plasmid and SGI1-C (Table 1). Interestingly, expression of FlhDC_SGI1_ yielded ca. 3-5-fold higher transfer frequency of either SGI1 or R16a than that of AcaCD. This demonstrates that FlhDC_SGI1_ is able to fulfill all the regulatory functions of AcaCD in the IncA/C transfer system and supports that the two activators have common targets.

**Table 1:** Trans complementation of conjugation deficiency of the R16aΔ*acaCD* mutant.

Donor	Transconjugant/recipient		Transconjugant/donor	
TG1Nal::SGI1-C/ R16aΔ*acaCD* +	R16aΔ*acaCD*	SGI1-C	R16aΔ*acaCD*	SGI1-C
**pJKI1036**^a^	<2.6±0.7×10^−8^	<2.6±0.7×10^−8^	<2.1±1.0×10^−8^	<2.1±1.0×10^−8^
**pJKI1038 (AcaCD)**	7.3±5.6×10^−4^	3.6±1.4×10^−3^	1.2±0.5×10^−3^	6.7±1.9×10^−2^
**pJKI1040 (FlhDC**_**SGI1**_**)**	5.5±1.5×10^−3^	1.0±0.3×10^−2^	5.3±1.7×10^−3^	9.9±3.0×10^−2^

^*a*^ pJKI1036 was used as negative control.

### Induction of the predicted AcaCD-dependent SGI1 promoters

Besides the AcaCD-binding site in P_*xis*_ four additional potential target sites have been predicted on SGI1 [16] in the upstream regions of orfs *S004*, *S005*, *S012* and *S018* (Fig 1A). To compare the promoter activity of these regions with that of P_*xis*_, upstream regions were inserted into β-galactosidase tester plasmids analogously to pJKI1003 (S3 Table). In these tester constructs, the putative promoter regions P_*S005*_, P_*S012*_ and P_*S018*_ began from the start codon of the annotated downstream orfs (according to sequence AF261825) and included the whole predicted AcaCD-binding site with 20 to 75 bp upstream flanking regions. As orf *S003* was previously suggested to be expressed from the putative promoter P_*S004*_ [16], a tester plasmid containing the upstream region of *S003* from the start codon of *S003* to the stop codon of *S005*, including the entire *S004*, was also created (pMSZ956). On the other hand, the predicted AcaCD-binding site in the upstream region of *S004* is located only 3 bp from the start codon of the annotated *S004* orf, which rules out the possibility of expression of the annotated S004 protein (designated here as S004_L_) from this putative AcaCD-dependent promoter. Comparing the spacing between the AcaCD-binding site and the start codon in AcaCD-driven promoters [16], we assumed that the translation of *S004*, if it is expressed at all, starts from the in-frame ATG codon located 66 bp downstream of the AcaCD-binding site (3474 bp in SGI1 sequence AF261825) and produces a shorter protein (designated as S004_S_). This assumption was further supported by the presence of a potential SD-box preceding this ATG codon. Accordingly, a tester plasmid, pMSZ965, containing the upstream region of the in-frame ATG located in *S004* to the stop codon of *S005*, was also constructed (S1 Fig).

Similarly to P_*xis*_, LacZ expression was undetectable from promoters P_*S005*_ and P_*S012*_ in the lack of activators. On the other hand, P_*S004*_ and P_*S018*_ had a measurable basal activity (5 and 15 units in β-galactosidase assay, respectively, Fig 1C). The low or undetectable expression levels observed for *xis*, *S003*, *S004* and *S005* without induction were in agreement with previously published data [22]. However, we found also undetectable expression level for *S012*, which was previously reported to show the most robust expression among these genes. Moreover, in case of *S018*, whose expression was reported to be similar to that of *S005* [22], we found the highest level of constitutive expression. Nevertheless, β-galactosidase assays proved that all putative promoter regions are inducible by either AcaCD or FlhDC_SGI1_, but the latter showed ca. 3-fold lower activity on each promoter (Fig 1C).

Induction of P_*S004*_, however, could only be detected with pMSZ965 where *lacZ* gene starts with the in-frame ATG of *S004*, but not with pMSZ956 where *lacZ* begins with the ATG of *S003*. The promoter activity in pMSZ956 was around the detection limit (2.3±0.7 units) independently of the presence of activators, which suggested that *S003* (*repA*) is not controlled by AcaCD or FlhDC _SGI1_ and expressed from a weak promoter probably located in *S004*. These results confirmed that all the predicted AcaCD-binding sites on SGI1 are parts of functional promoters that can be induced by either AcaCD or FlhDC_SGI1_, and indicate that both activators can potentially participate in the regulation of the genes located downstream of these promoters *in vivo*.

### Determination of TSS of AcaCD-controlled SGI1 genes

**Fig 2:**
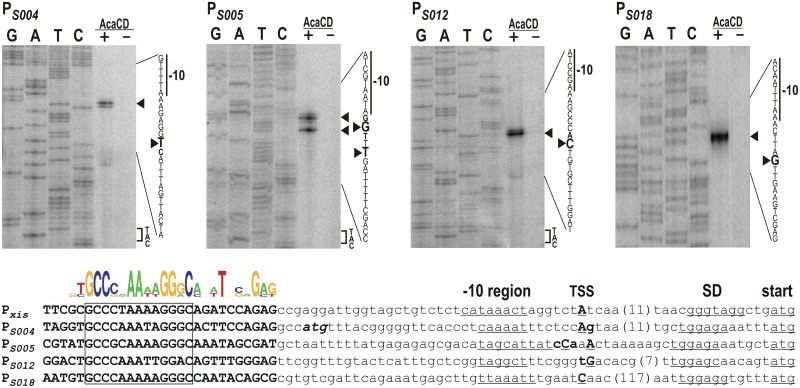
Determination of TSSs of AcaCD/FlhDC_SGI1_-responsive SGI1 genes. Primer extension reactions were performed using primer pUCfor21 and total RNA purified from *E*. *coli* TG1 carrying tester plasmids pMSZ965 (P_*S004*_), pMSZ953 (P_*S005*_), pMSZ954 (P_*S012*_) and pMSZ955 (P_*S018*_) +/- the AcaCD producer plasmid pJKI888 (lanes + and -). Lanes G, A, T, C: Sanger sequencing reactions obtained using pUCfor21 and the appropriate tester plasmid as template DNA. Arrowheads point to the base on the non-transcribed strand corresponding to the TSS on the sense strand. The putative -10 box and the start codon (if applicable) are indicated. The presence (+) or absence (-) of AcaCD is shown. The sequence alignment of the five AcaCD-dependent promoter regions are shown below the images. The putative -10 box, SD-box and the start codon are underlined. TSSs detected are shown as bold, underlined uppercases indicate the most frequent start sites. The predicted AcaCD-binding sites are shown as bold uppercase, the conserved core regions are boxed. Conserved positions are represented by sequence logo. The start codon of the annotated *S004* orf is indicated as bold italics.

After demonstrating the AcaCD/FlhDC_SGI1_-dependent activation of P_*S004*_, P_*S005*_, P_*S012*_ and P_*S018*_ promoters, the transcription start sites (TSS) were determined using the appropriate tester plasmids in primer extension assays. In case of all four promoters, extension reaction was observed only when the activator AcaCD was expressed from a compatible plasmid (as it was shown that AcaCD has stronger activation effect, only this activator was used in primer extension assays) (Fig 2). The TSS mapping showed that the *S004* transcript begins 30 bp upstream of the previously predicted in-frame ATG codon, however a weaker signal was also detected at the 29 bp position suggesting that this mRNA has alternative start sites at these neighboring positions. This result, as expected, excludes the usage of the ATG determined by the original *S004* annotation and supports our hypothesis that the orf is more probably expressed from the in-frame ATG located at an optimal distance from the AcaCD-binding site. The next AcaCD-controlled transcript encoding S005 (TraN) also showed unambiguous TSS. The strongest signals showed that the transcript begins 22 or 25 bp upstream from the start codon of *S005*, however, weaker signals could be seen at 24 and 26 bp positions, as well. Majority of the *S012* transcripts begins 26 bp upstream of the start codon, but a small fraction starts at 27 bp. The longest 5’ UTR was observed in *S018* transcript where TSS was detected at 135 bp from the ATG. Although a relatively high basal activity was measured in the β-galactosidase assay for P_*S018*_, the TSS of this constitutively expressed transcript was not detectable (see P_*S018*_ panel, lane AcaCD—in Fig 2). This suggests that a weak constitutive promoter may also be located elsewhere in the upstream region of *S018*. While all four promoters have a -10 box 5–7 bp upstream of TSSs, no obvious -35 boxes can be found. Instead, the 3’ end of the conserved region of AcaCD binding sites are located at the expected -35 position as was observed in P_*xis*_ [18] and other AcaCD-dependent promoters in IncA/C plasmids [16].

### S004_S_ has a negative effect on bacterial growth

Since both the β-galactosidase assay and TSS mapping suggested that the translation of *S004* starts at a different position than was predicted by the annotation of SGI1 orfs (GenBank: AF261825), we examined whether the expression of *S004* has any detectable effects on the growth of host bacteria. According to the original annotation, orf *S004* encodes 96 amino acids, while our results suggested that it might be expressed from an in-frame ATG as a shorter (75 aa) polypeptide (S004_L_ and S004_S_ referring to “longer” and “shorter” proteins, respectively). The two orfs beginning with the different ATGs were placed under the control of P_*tac*_ (pJKI1049, pJKI1050) and their effect on bacterial growth was monitored by determination of growth curves. For comparison, an analogous plasmid expressing S003 and the empty vector as negative control were also applied (pJKI1048 and pGMY8, see S3 Table). S003 was chosen as it is a RepA homologue, which has no cognate replication origin in the host strain and probably has no function. Thus, its expression probably has a metabolic load on the host bacteria without additional toxic effects.

**Fig 3:**
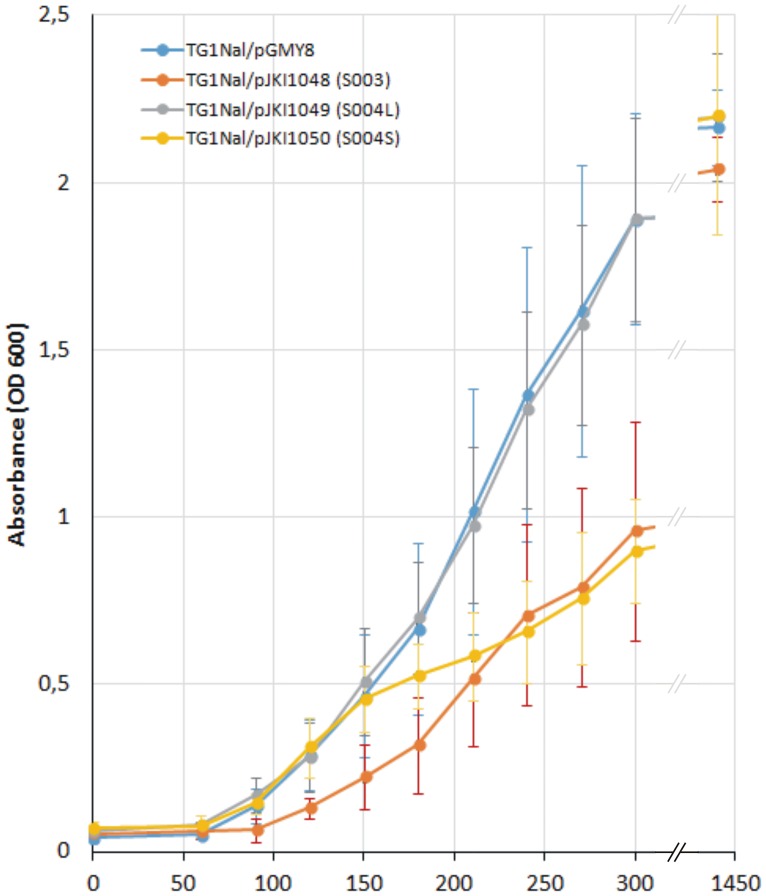
Growth curves of TG1Nal strains harboring the plasmids expressing S003, S004_L_ and S004_S_ proteins. OD_600_ values are represented as the means of six parallels. The empty plasmid vector pGMY8 was used as a negative control.

The proper expression of proteins S004_L_ (11.3 kDa), S004_S_ (8.8 kDa) and S003 (35.3 kDa) was tested by PAGE of total protein samples obtained from plasmid-bearing strains, which showed that S004_L_ appears to be expressed in lower level compared to S004_S_ and S003 (S2 Fig). The expression plasmids then were introduced to *E*. *coli* TG1Nal host strain and the growth curves were recorded under inducing conditions. Although OD_600_ of each culture reached 2.0–2.2 after 24 hours incubation, their growth showed significant difference in the first 5 hours (Fig 3). While expression of S004_L_ had no detectable effect on the growth rate compared to the negative control, production of S004s retarded growth to a similar extent as did production of the S003 protein which has a molecular mass four times that of S004s. Blastp database search revealed that S004 contains an HTH motif (36–90 aa region) related to HTH of MerR regulatory proteins and shows 38–42% identity and 59–64% similarity (mostly in this region) to AlpA phage regulatory proteins. Although the HTH motif is present in both possible variants of S004, the growth rates of strains expressing them support that S004_S_ may have more significant biological effects and S004_L_, if expressed at all, is probably inactive and/or degraded rapidly.

## Conclusions

We have demonstrated that AcaCD binding sites in SGI1 predicted previously [16] are located in functional promoters that can be induced by the IncA/C plasmid-encoded AcaCD as well as by the SGI1-encoded FlhDC_SGI1_ master activators. Similarly to P_*xis*_, the four additional SGI1 promoters analysed in this work also lack obvious -35 promoter box and their -10 box is located 22–24 bp downstream of the AcaCD binding site. In general, their promoter profile appears to be consistent with promoters of the class II-type activation pathway [23].

The two activators are close relatives as they share 79 and 46% identity in the amino acid sequence of C and D subunits, respectively [18]. Despite their sequence divergence, FlhDC_SGI1_ is able to act similarly on the AcaCD-dependent SGI1 promoters, and can also substitute for AcaCD in the regulation of the whole conjugation apparatus of the IncA/C plasmids. This compatibility of FlhDC_SGI1_ and AcaCD may raise the possibility of complex interactions between SGI1 and IncA/C helper plasmids when they occur in the same cell. However, the functions of FlhDC_SGI1_ and the AcaCD-responsive SGI1 genes have not yet been established in detail with the exception of *xis*. P_*xis*_ appears to be a sensor of the helper plasmid entry as the plasmid-borne activator induces *xis* expression through binding to P_*xis*_, which leads to triggering SGI1 excision [18]. We have shown previously that the deletion removing the orfs from *S005* to *S012* has no significant effect on the SGI1 conjugation frequency, indicating that neither FlhDC_SGI1_ (encoded by orfs *S007-S006*) nor the AcaCD-responsive *S004* and the putative conjugation-related genes *S005* (*traN*), *S011* (*traG*) and *S012* (*traH*), are required for efficient mobilization of SGI1. Similarly, deletion of the *S013-S018* region containing short orfs without known functions has no effect on the transfer frequency under laboratory circumstances (G. M. unpublished data). A possible explanation can be that *flhDC*_SGI1_ and its target promoters are remnants from the earlier evolution of the island. Accordingly, the ancient SGI1 could possess the whole conjugation apparatus and its regulatory functions, like SXT elements or the related IncA/C plasmids [24]. In this case, the similarity of the plasmid- and SGI1-encoded activators and their target sequences, especially in case of P_*xis*_, might have contributed to the evolution of the recent form of SGI1 that has lost the ability for self-transfer but can efficiently hijack IncA/C plasmids. Although few deletions can be found in AcaCD-controlled genes (Δ*xis* in *Acinetobacter baumannii* strain D4, GenBank: KP054476.2; Δ*S004* in *Shewanella* sp. W3-18-1, GenBank: CP000503.1; Δ*S005* in several *Proteus mirabilis* strains), the whole region is highly conserved at the DNA sequence level (85–100% identity) even in distant relatives of SGI1, which might imply some kind of selection for the integrity of these functions.

## Supporting Information

S1 Fig Linear map of *S003-S005* region of SGI1. Orfs are represented by purple arrows (*S003* and *S005* are not shown as full length orfs). The elements of the AcaCD-responsive promoter are indicated by grey boxes (abbreviations are: TSS *S004*_S_, transcription start site of *S004*_S_; -10, -10 box). The promoter regions inserted into pMSZ956 and pMSZ965 tester plasmids are shown below the graph.
(TIF)

S2 Fig SDS PAGE for expression of S003, S004_L_ and S004_S_. Two parallel colonies harboring the expression vectors were used in the assays. Lanes—and + show the total proteins obtained from non-induced and induced cultures, respectively.
(TIF)

S1 Table Oligonucleotide primers used in this study. (DOC)

S2 Table Specifications of bacterial strains used in this study. (DOC)

S3 Table List of plasmids. (DOC)

S1 Text Construction of plasmids. (DOC)
