# Supplementary figures and images for: Determination and Analysis of the Putative AcaCD-Responsive Promoters of Salmonella Genomic Island 1
Source: PLoS One. 2016 Oct 11;11(10):e0164561. doi: 10.1371/journal.pone.0164561 (PMC5058578; doi:10.1371/journal.pone.0164561)

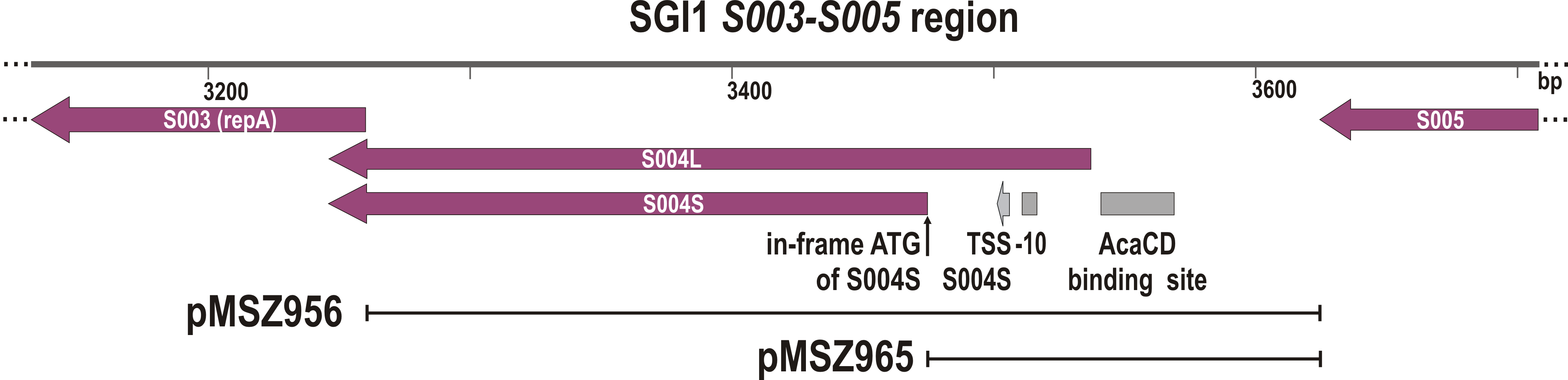

Supplement: S1 Fig — Orfs are represented by purple arrows (S003 and S005 are not shown as full length orfs). The elements of the AcaCD-responsive promoter are indicated by grey boxes (abbreviations are: TSS S004S, transcription start site of S004S; -10, -10 box). The promoter regions inserted into pMSZ956 and pMSZ965 tester plasmids are shown below the graph. (TIF) [file pone.0164561.s001.TIF]

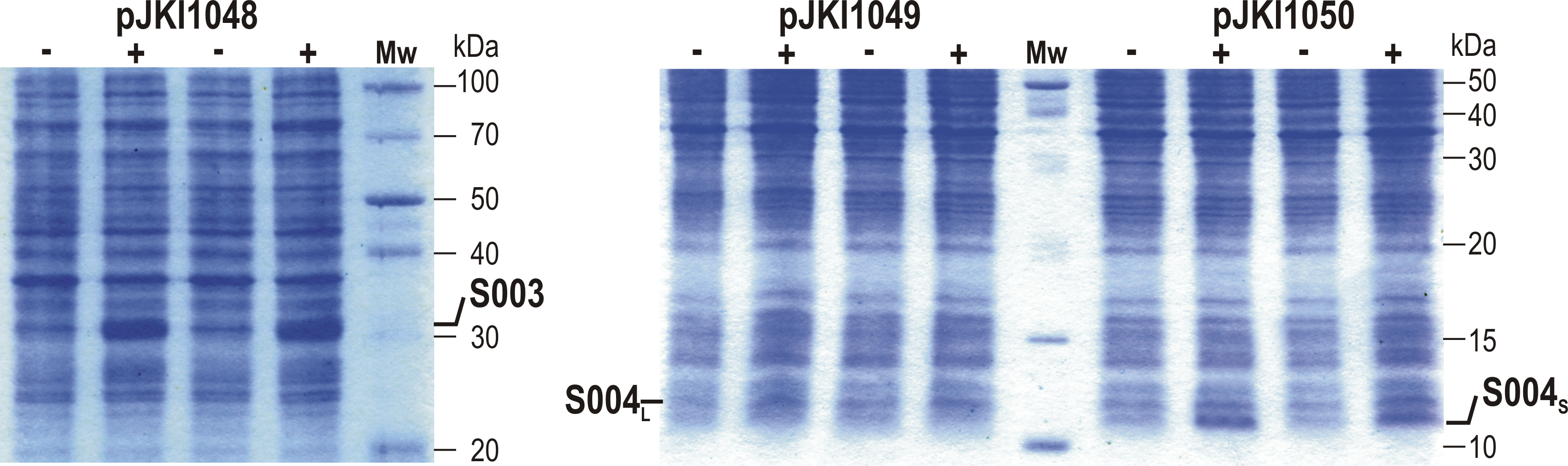

Supplement: S2 Fig — Two parallel colonies harboring the expression vectors were used in the assays. Lanes—and + show the total proteins obtained from non-induced and induced cultures, respectively. (TIF) [file pone.0164561.s002.TIF]
