# Supplementary material for: Determination and Analysis of the Putative AcaCD-Responsive Promoters of Salmonella Genomic Island 1
Source: PLoS One. 2016 Oct 11;11(10):e0164561. doi: 10.1371/journal.pone.0164561 (PMC5058578; doi:10.1371/journal.pone.0164561)
Supplement: S1 Table — (DOC) [file pone.0164561.s003.doc]

**S1 Table. Oligonucleotide primers used in this study.**

| **Name** | **Sequence (5’→3’)** | **References** |
| --- | --- | --- |
| S005promfor_Nc | ggccatggcatttctccagctttttagtttg | this work |
| S005promrev_P | ctctgcagctgcctgaatacatccg | this work |
| S012promfor_Nc | tcccatggtgttgctccaataggtttcgtgtc | this work |
| S012promrev_P | tgctgcagcaaagttgccgacattattcaatg | this work |
| S018promfor_Nc | gtccatggacacctccaattagttggaattg | this work |
| S018promrev_P | aactgcagcggctctgctgattaaaagcc | this work |
| S003promfor_Nc | ctccatggcttaccctgcgggaatc | this work |
| S003promrev_X | tttctagaggcaatggtggttgatgtttatc | this work |
| S004promfor_Nc | gcccatggatttctccagcatcatcattgat | this work |
| S003_Ndefor | taacatatgaagccatttagccaactacttgc | this work |
| S003_BXhrev | aactcgagggatcctcaattacggtatggaatcggtgg | this work |
| S004_Ndefor1 | gagcatatgtttacgggggttcaccctcaaaatttc | this work |
| S004_Ndefor2 | aaacatatgcagcaaactttaaatgaaaaagattac | this work |
| S004_BXhrev | ttctcgagggatcctaaatggcttcattacttaccctgcg | this work |
| S007_Ndfor | ttcatatgtatgccttagagccgttagaacg | this work |
| S006_Bamrev | ttggatcctcaggcagcttttgagacccggc | [1] |
| pUCfor21 | cagggttttcccagtcacgac | [2] |

Restriction sites are underlined.

**References**

1. Kiss J, Papp PP, Szabó M, Farkas T, Murányi G, Szakállas E, et al. The master regulator of IncA/C plasmids is recognized by the Salmonella Genomic island SGI1 as a signal for excision and conjugal transfer. Nucleic Acids Res. 2015;43: 8735–8745. doi:10.1093/nar/gkv758

2. Kiss J, Olasz F. Formation and transposition of the covalently closed IS 30 circle : the relation between tandem dimers and monomeric circles. Mol Microbiol. 1999;34: 37–52.
