# Supplementary material for: Determination and Analysis of the Putative AcaCD-Responsive Promoters of Salmonella Genomic Island 1
Source: PLoS One. 2016 Oct 11;11(10):e0164561. doi: 10.1371/journal.pone.0164561 (PMC5058578; doi:10.1371/journal.pone.0164561)
Supplement: S2 Table — (DOC) [file pone.0164561.s004.doc]

**S2 Table. Specifications of bacterial strains used in this study.**

| ***E. coli* strains** | **Genotype or relevant features** | **References** |
| --- | --- | --- |
| TG1Nal | Nal^R^ derivative of TG1 | [1] |
| TG1Nal::SGI1-C | Nal^R^Sm^R^Sp^R^Su^R^, SGI1-C transconjugant of TG1Nal. Single copy of SGI1-C is integrated in attB (*trmE*). | [2] |
| TG1Nal::SGI1-C/ R16aΔ*acaCD* | TG1Nal::SGI1-C containing *acaCD* KO mutant R16a, Nal^R^Sm^R^Sp^R^Su^R^Km^R^Ap^R^ | this work |
| TG90 | *pcn B80 zad*::Tn*10* (Tc^R^) derivative of TG1 | [3] |
| Tuner (DE3) | F^-^ *ompT hsdS_B_(r_B_^-^ m_B_^-^) gal dcm lacY1* (DE3) | Novagen |

**References**

1. Kiss J, Nagy B, Olasz F. Stability, entrapment and variant formation of Salmonella genomic island 1. PLoS One. 2012;7: e32497. doi:10.1371/journal.pone.0032497

2. Kiss J, Papp PP, Szabó M, Farkas T, Murányi G, Szakállas E, et al. The master regulator of IncA/C plasmids is recognized by the Salmonella Genomic island SGI1 as a signal for excision and conjugal transfer. Nucleic Acids Res. 2015;43: 8735–8745. doi:10.1093/nar/gkv758

3. Gonzy-Treboul G, Karmazyn-Campelli C, Stragier P. Developmental regulation of transcription of the Bacillus subtilis ftsAZ operon. J Mol Biol. 1992;224: 967–979. doi:10.1016/0022-2836(92)90463-T
