# Supplementary material for: Determination and Analysis of the Putative AcaCD-Responsive Promoters of Salmonella Genomic Island 1
Source: PLoS One. 2016 Oct 11;11(10):e0164561. doi: 10.1371/journal.pone.0164561 (PMC5058578; doi:10.1371/journal.pone.0164561)
Supplement: S3 Table — (DOC) [file pone.0164561.s005.doc]

**S3 Table. List of plasmids.**

| **Name** | **Relevant features** | **References** |
| --- | --- | --- |
| pJKI88 | Km^R^, p15A cloning vector | [1] |
| pJKI391 | pJKI88 derivative expression vector containing P*_tac_* followed by a MCS and *lacI^q^* gene | [2] |
| pJKI596 | pBluescript SK vector containing Gm^R^ gene | this work |
| pJKI888 | pJKI391 derivative containing the *acaCD* genes of R55 under the control of P*_tac_* promoter | [2] |
| pJKI990 | pJKI861 derivative cloning vector for β-gal assays supplemented with the *rrnB* terminator | [2] |
| pJKI1003 | pJKI990 derivative tester plasmid containing P*_xis_* region (1947-2306 bp) | [2] |
| pJKI1013 | pJKI990 derivative tester plasmid containing proximal P*_xis_* fragment (1947-2052 bp) | [2] |
| pJKI1014 | pJKI990 derivative tester plasmid containing proximal P*_xis_* fragment (1947-2043 bp) | [2] |
| pJKI1016 | pJKI990 derivative tester plasmid containing proximal P*_xis_* fragment (1947-2012 bp) | [2] |
| pJKI1021 | Sm/Sp^R^ derivative of pJKI391 containing ORF001 of R55 under the control of P*_tac_* and *rrnB* terminator | this work |
| pJKI1036 | Gm^R^ derivative of pJKI391 expression vector | this work |
| pJKI1038 | Gm^R^ derivative of pJKI888 expression vector | this work |
| pJKI1040 | Gm^R^ derivative of pGMY6 expression vector | this work |
| pJKI1042 | Bluescript SK vector containing ORF *S003* | this work |
| pJKI1043 | Bluescript SK vector containing ORF *S004*_L_ | this work |
| pJKI1044 | Bluescript SK vector containing ORF *S004*_S_ | this work |
| pJKI1048 | Sm/Sp^R^ derivative of pJKI391 containing ORF *S003* of SGI1 under the control of P_tac_ and *rrnB* terminator | this work |
| pJKI1049 | Sm/Sp^R^ derivative of pJKI391 containing ORF *S004*_L_ of SGI1 under the control of P*_tac_* and *rrnB* terminator | this work |
| pJKI1050 | Sm/Sp^R^ derivative of pJKI391 containing ORF *S004*_S_ of SGI1 under the control of P*_tac_* and *rrnB* terminator | this work |
| pMSZ953 | pJKI990 derivative tester plasmid containing P*_S005_* region of SGI1 (6385-6490 bp) | this work |
| pMSZ954 | pJKI990 derivative tester plasmid containing P*_S012_* region of SGI1 (13421-13564 bp) | this work |
| pMSZ955 | pJKI990 derivative tester plasmid containing P*_S018_* region of SGI1 (16189-16462 bp) | this work |
| pMSZ956 | pJKI990 derivative tester plasmid containing P*_S003_* region of SGI1 (3261-3639 bp) | this work |
| pMSZ965 | pJKI990 derivative tester plasmid containing P*_S004_* region of SGI1 (3474-3639 bp) | this work |
| pGMY6 | pJKI391 derivative containing the *flhDC_SGI1_* genes of SGI1 under the control of P*_tac_* promoter | this work |
| pGMY8 | expression vector, Sm/Sp^R^ derivativeof pJKI391 | this work |
| R16aΔ*acaCD* | *acaCD* KO mutant of IncA/C plasmid R16a, Ap^R^Km^R^Su^R^ | [2] |

**References**

1. Kiss J, Olasz F. Formation and transposition of the covalently closed IS 30 circle : the relation between tandem dimers and monomeric circles. Mol Microbiol. 1999;34: 37–52.

2. Kiss J, Papp PP, Szabó M, Farkas T, Murányi G, Szakállas E, et al. The master regulator of IncA/C plasmids is recognized by the Salmonella Genomic island SGI1 as a signal for excision and conjugal transfer. Nucleic Acids Res. 2015;43: 8735–8745. doi:10.1093/nar/gkv758
