# Supplementary material for: Determination and Analysis of the Putative AcaCD-Responsive Promoters of Salmonella Genomic Island 1
Source: PLoS One. 2016 Oct 11;11(10):e0164561. doi: 10.1371/journal.pone.0164561 (PMC5058578; doi:10.1371/journal.pone.0164561)
Supplement: S1 Text — (DOC) [file pone.0164561.s006.doc]

**S1 Text. Construction of plasmids.**

For complementation tests, the Km^R^ resistance marker of pJKI888[1] and pGMY6 expressing *acaCD* and *flhDC_SGI1_* genes, respectively, and the expression vector pJKI391[1] was replaced by the Gm^R^ gene. The Gm^R^ cassette was amplified from pJQ200SK [2] with primers GmRforSX and GmRrevSX, and then was ligated into the SmaI site of pBluescript SK resulting in pJKI596. As the next step, it was cloned as a HincII fragment from pJKI596 into the unique SmaI site within the Km^R^ gene of the three expression vectors resulting pJKI1038, pJKI1040 and pJKI1036 plasmids, respectively.

For primer extension analyses and β-galactosidase assays, tester plasmids were constructed by cloning the promoter regions of interest into pJKI990[1]. P*_S005_*, P*_S012_*, and P*_S018_* were amplified with primer pairs S005promfor_Nc-S005promrev_P, S012promfor_Nc-S012promrev_P and S018promfor_Nc-S018promrev_P and the amplicons were cloned into the NcoI-PstI site of pJKI990, resulting in pMSZ953, pMSZ954 and pMSZ955, respectively. P*_S003_* and P*_S004_* were amplified using primer pairs S003promfor_Nc-S003promrev_X and S004promfor_Nc-S003promrev_X and cloned into the NcoI-XbaI site of pJKI990, resulting in pMSZ956 and pMSZ965, respectively.

The p15a-based expression vectors containing ORFs *S003*, *S004*_L_ and *S004*_S_ were constructed as follows. The three ORFs were amplified with the primers S003_Ndefor-S003_BXhrev, S004_Ndefor1-S004_BXhrev and S004_Ndefor2-S004_BXhrev, respectively, and cloned into the SmaI site of pBluescriptSK, resulting in pJKI1042, pJKI1043 and pJKI1044. After sequencing the inserts, the three ORFs were cloned as NdeI-BamHI fragments into the Sm/Sp^R^ derivative of pJKI391 (pJKI1021), between the P*_tac_* promoter and the *rrnB* terminator (replacing the original insert), resulting in pJKI1048, pJKI1049 and pJKI1050, respectively. For the construction of negative control plasmid pGMY8, pJKI1048 was digested with NdeI-BamHI, and the vector was ligated after filling-in the sticky ends with Klenow polymerase (ORF *S003* was excised).

To construct the FlhDC_SGI1_*-*producer plasmid pGMY6, ORFs *S007-S006* of SGI1 (7628-6481 bp) were amplified using primers S007_Ndfor-S006_Bamrev. The amplicon was cloned into the NdeI-BamHI site of pJKI391 in two pieces (since *S007* contains an NdeI site) resulting in pGMY5 (carrying incomplete *S007*) and pGMY6 containing full length *S007* and *S006* under the control of P*_tac_*.

**References**

1. Kiss J, Papp PP, Szabó M, Farkas T, Murányi G, Szakállas E, et al. The master regulator of IncA/C plasmids is recognized by the Salmonella Genomic island SGI1 as a signal for excision and conjugal transfer. Nucleic Acids Res. 2015;43: 8735–8745. doi:10.1093/nar/gkv758

2. Quandt J, Hynes MF. Versatile suicide vectors which allow direct selection for gene replacement in gram-negative bacteria. Gene. 1993;127: 15–21. doi:10.1016/0378-1119(93)90611-6
